# Whole-genome analysis suggesting probiotic potential and safety properties of *Pediococcus pentosaceus* DSPZPP1, a promising LAB strain isolated from traditional fermented sausages of the Basilicata region (Southern Italy)

**DOI:** 10.3389/fmicb.2024.1268216

**Published:** 2024-04-04

**Authors:** Madhura S. Tathode, Maria Grazia Bonomo, Silvia Zappavigna, Stefania Mirela Mang, Marco Bocchetti, Ippolito Camele, Michele Caraglia, Giovanni Salzano

**Affiliations:** ^1^Department of Precision Medicine, University of Campania “Luigi Vanvitelli”, Naples, Italy; ^2^Department of Science, Università degli Studi della Basilicata, Potenza, Italy; ^3^Spinoff TNcKILLERS, Potenza, Italy; ^4^School of Agricultural, Forestry, Food and Environmental Sciences (SAFE), Università degli Studi della Basilicata, Potenza, Italy; ^5^Laboratory of Molecular and Precision Oncology, Biogem Scarl, Institute of Genetic Research, Ariano Irpino, Italy

**Keywords:** *Pediococcus pentosaceus*, probiotics, whole genome sequencing, antimicrobial resistance, bacteriocins

## Abstract

**Introduction:**

Many lactic acid bacteria (LAB) strains are currently gaining attention in the food industry and various biological applications because of their harmless and functional properties. Given the growing consumer demand for safe food, further research into potential probiotic bacteria is beneficial. Therefore, we aimed to characterize *Pediococcus pentosaceus* DSPZPP1, a LAB strain isolated from traditional fermented sausages from the Basilicata region of Southern Italy.

**Methods:**

In this study, we analyzed the whole genome of the *P. pentosaceus* DSPZPP1 strain and performed *in silico* characterization to evaluate its applicability for probiotics and use in the food industry.

**Results and Discussion:**

The whole-genome assembly and functional annotations revealed many interesting characteristics of the DSPZPP1 strain. Sequencing raw reads were assembled into a draft genome of size 1,891,398 bp, with a G + C content of 37.3%. Functional annotation identified 1930 protein-encoding genes and 58 RNAs including tRNA, tmRNA, and 16S, 23S, and 5S rRNAs. The analysis shows the presence of genes that encode water-soluble B-group vitamins such as biotin, folate, coenzyme A, and riboflavin. Furthermore, the analysis revealed that the DSPZPP1 strain can synthesize class II bacteriocin, penocin A, adding importance to the food industry for bio-enriched food. The DSPZPP1 genome does not show the presence of plasmids, and no genes associated with antimicrobial resistance and virulence were found. In addition, two intact bacteriophages were identified. Importantly, the lowest probability value in pathogenicity analysis indicates that this strain is non-pathogenic to humans. 16 s rRNA-based phylogenetic analysis and comparative analysis based on ANI and Tetra reveal that the DSPZPP1 strain shares the closest evolutionary relationship with *P. pentosaceus* DSM 20336 and other *Pediococcus* strains. Analysis of carbohydrate active enzymes (CAZymes) identified glycosyl transferases (GT) as a main class of enzymes followed by glycoside hydrolases (GH). Our study shows several interesting characteristics of the isolated DSPZPP1 strain from fermented Italian sausages, suggesting its potential use as a promising probiotic candidate and making it more appropriate for selection as a future additive in biopreservation.

## 1 Introduction

LAB include a group of Gram-positive rods and cocci, non-sporing, and catalase-negative microorganisms that are of great importance in fermented food industries for their biochemical, physiological, and genetic features (Shukla and Goyal, 2014). LAB are widely present in foods and used as biopreservatives as well as aroma, texture, and flavor enhancers. Various studies have demonstrated the close connection between the LAB properties and their ability to produce, through fermentation, a wide range of sugars and metabolites, such as lactic and acetic acid, ethanol, acetone, diacetyl, exopolysaccharides, specific proteases, and protein antimicrobials, called bacteriocins (Papagianni, 2012; Cotter et al., 2013; Gudiña et al., 2015; Barbosa et al., 2017; Zommiti et al., 2018). Screening for beneficial bacteria from complex microbial communities is becoming more common. Of the many known microorganisms, few have been tested to determine their characteristics and applications. Among these, microorganisms called probiotics are receiving much interest due to their beneficial effects on plants, animals, humans, and foods, and there is an increasing effort to identify other new bacteria that may be both functional and harmless.

Currently, only a limited number of probiotics are used for the daily maintenance of intestinal microbial balance and in clinical treatment. Among them, some LABs have proved worthy of exploration in human health promotion (Jiang et al., 2021).

Although several species of *Lactobacillus*, *Bifidobacterium,* and *Clostridium* possess potent probiotic characteristics, these strains are not sufficient for the various needs of humans and industry (Min et al., 2017). Therefore, the identification of additional bacterial species is required to enrich the application spectrum of probiotics and their applications in food and agriculture (Jiang et al., 2021). Several studies have tested and used bacterial species belonging to the genus *Pediococcus* that have highlighted the importance of *Pediococcus* in the field of probiotics, indicating that new strains, isolated from different food matrices and belonging to this genus, can play a key role in the development of new functional foods (Martino et al., 2013; Vidhyasagar and Jeevaratnam, 2013; Savedboworn et al., 2014; Dubey et al., 2015; Chen et al., 2017; Zommiti et al., 2018; Todhanakasem et al., 2020; Hu et al., 2021). Numerous studies have reported that strains of *Pediococcus (P.) pentosaceus* are frequently isolated from various food sources and biotopes encompassing plant materials, bacterial ripened cheese, beverages, pickles, wine, dairy, and meat products, with a potent role as starter cultures involved in the manufacturing of fermented foods (Halami et al., 2005; Midha et al., 2012; García-Ruiz et al., 2014; Lv et al., 2014; Carafa et al., 2015; Ilavenil et al., 2016; Zommiti et al., 2018).

*Pediococcus pentosaceus*, belonging to the family *Lactobacillaceae*, genus *Pediococcus*, is a species of cocci-shaped LAB in pairs or quadruplets and is a non-motile facultative anaerobic Gram-positive bacterium (Chen et al., 2020). Previous studies have shown that *P. pentosaceus* occurs naturally in fermented products and may have an active function in product quality, food safety, and production efficiency (Balakrishnan and Agrawal, 2014; Jang et al., 2015; Gong and Qi, 2020; Montemurro et al., 2020; Jiang et al., 2021; Xu et al., 2021). Furthermore, different research has reported that *P. pentosaceus* has probiotic functions including anti-inflammatory, anti-cancer, antioxidant, detoxifying, and cholesterol-lowering activities (Zhao et al., 2012; Thirabunyanon and Hongwittayakorn, 2013; Sellamani et al., 2016; Asami et al., 2017; Kim et al., 2019). However, the use of LAB does not support the different needs in food processing, so the demand for more types of probiotic strains is growing and *P. pentosaceus* represents a good candidate due to its application value and probiotic effect attracting great interest by the food industry (Qi et al., 2021). Researchers from various countries have studied the probiotic potential of *P. pentosaceus* in local foods to determine the best use of *P. pentosaceus* in the food industry, maintaining its probiotic effect in the human body (Shukla and Goyal, 2014; Sellamani et al., 2016; Nanasombat et al., 2017; Ghosh et al., 2019).

For this reason, in many studies (Cocolin et al., 2004; Ammor et al., 2005; Comi et al., 2005; Aymerich et al., 2006; Baruzzi et al., 2006; Bonomo et al., 2008), the microbial diversity identification and characterization were assessed. In Italy, different typical meat products, such as the traditional fermented sausages of the Basilicata region, were analyzed to identify suitable starter cultures for use in the production process and necessary to guarantee food safety and to standardize product properties (Lebert et al., 2007). It has been observed that artisanal products have greater quality than sausages from controlled fermentations; commercial starter cultures are often not able to compete well with the house flora so their use often results in the loss of desirable sensory properties (Bonomo et al., 2008, 2013). Therefore, the tendency has been to select appropriate cultures from indigenous microorganisms that are more competitive and well adapted to the particular product and the specific production technology, and with high metabolic capacities, which can beneficially affect product quality and safety, preserving their typicity (Ammor et al., 2005; Drosinos et al., 2005; Aymerich et al., 2006; Leroy et al., 2006; Bonomo et al., 2008).

In a previous study, Parente et al. (2001) evaluated the phenotypic diversity of lactic acid bacteria (LAB) populations in traditional fermented sausages produced in artisanal and industrial plants in Basilicata (Southern Italy). In total, 414 LAB cultures were isolated from samples of sausages at different stages of ripening and a set of 28 tests was used to classify and identify the isolates; 50% of the isolates were identified as *Lactobacillus (L.) sakei*, 22% as *Pediococcus pentosaceus*, and 7% as *Leuconostoc* spp. and other *Lactobacillus* spp., were present in lower percentages.

Among these tested isolates, there is also our strain (*P. pentosaceus* DSPZPP1) belonging to a cluster that proved interesting properties. So, further analyses were conducted on some strains selected among these isolates investigated. In the previous study by Bonomo et al. (2008), 49 lactic acid bacteria (LAB) isolated from traditional fermented sausages of the Basilicata region were molecularly investigated for taxonomic identification at species and strain levels and technologically characterized on the basis of salt tolerance, acid production ability, growth at different temperatures, proteolytic, antimicrobial, and nitrate reductase activities. *Lactobacillus sakei* was the predominant species (67%) followed by *Pediococcus pentosaceus* (16%), *Leuconostoc carnosum* (8%), *Lb. plantarum* (4%), *Lb. brevis* (2%), and *Leuc. pseudomesenteroides* (2%). The technological characterization was conducted to highlight the primary purpose of LAB starter cultures used in meat fermentation, which is to enhance product safety and extend shelf life. LAB achieve this by reducing pH, growing, and being competitive in the conditions of sausage manufacturing, as well as by producing antimicrobial compounds. Moreover, a no-hierarchical cluster analysis by k-means procedure that classified strains in eight clusters on the basis of their technological profile was carried out. One of the three clusters that included the strains with the most interesting technological characteristics was the number 7 which included our strain *P. pentosaceus* DSPZPP1 (already labeled DBPZ0346). These strains presented a good growth, even in the presence of salt and nitrite. They also demonstrated a strong acidifying ability, moderate proteolytic and nitrate reductase activities, and displayed an antimicrobial activity against *Staphylococcus epidermidis* DSM1798 and *Kocuria* var*ians* LMG14231 strains (Bonomo et al., 2008). Therefore, based on the information on the abilities of this bacterial strain, we decided to deepen the study of the *P. pentosaceus* DSPZPP1 strain to better understand in a more precise and in-depth way the characteristics of the strain, which can then be used for various applications. This study arises from the need to use different and customized screening methods for *P. pentosaceus* based on individual characteristics and different applications of the product (Jiang et al., 2020) and from the limitation related to the source and species of *P. pentosaceus*, which pushes us to select strains with probiotic activities from different sources. Although numerous studies have found that *P. pentosaceus* has very important uses, systematic and relevant studies on the genetic research and application of *P. pentosaceus* are still lacking (Qi et al., 2021).

In this study, the whole-genome assembly and functional annotations of *P. pentosaceus* DSPZPP1 strain were performed to gain a thorough understanding of the functions and related mechanisms of the strain and to reveal interesting characteristics of the strain, suggesting its potential use as a promising probiotic candidate and making it more appropriate for selection as a future additive in biopreservation.

## 2 Materials and methods

### 2.1 Bacterial strain and culture conditions

The *P. pentosaceus* DSPZPP1 strain was isolated from traditional fermented sausage (Basilicata region, Southern Italy) (Parente et al., 2001; Bonomo et al., 2008). The strain was maintained as freeze-dried stocks in reconstituted (11% w/v) skim milk, containing 0.1% (w/v) ascorbic acid in the culture collection of the Microorganisms Genetics Laboratory of the Department of Sciences, University of Basilicata (Potenza, Italy), and routinely propagated in MRS broth at 37°C for 16 h before the analyses.

### 2.2 Whole-genome sequencing and annotation

Genomic DNA was extracted from 5 mL of late-exponential phase (A600 = 0.7–0.8) bacterial culture using The quick-DNATM fungal/bacterial MiniPrep Kit, provided By Zymo research Europe GmbH (Freiburg, Germany). DNA integrity Was confirmed through agarose electrophoresis, while Its concentration and purity were assessed By readings obtained using a NanoDrop ND-1000 spectrophotometer (NanoDrop technologies Inc., Wilmington, USA). The indexed libraries were generated using The Illumina Nextera DNA flex library preparation Kit. A whole-genome sequencing experiment was performed on The Illumina NextSeq 500 platform with a 2x150bp paired-end module at The biogem research institute, medicinal investigational research—M.I.R. (Ariano Irpino-AV, Italy). Prior quality control assessment Is performed using FASTQC v0.12.1.^1^ High-quality (HQ) filtered reads from The Raw data were obtained using NGS QC toolkit v2.3.3 (Patel and Jain, 2012) with The quality score cutoff ≥Q20. Primary assembly (contigs) were generated from high-quality filtered reads using short read *de-novo* assembler VELVET v1.2.10 (Zerbino, 2010). Scaffolding of pre-assembled contigs Was performed using SSPACE v2.0 (Boetzer et al., 2011). Output scaffolds were further anchored using *Pediococcus* representative genome *P. pentosaceus* ATCC 25745 [NCBI RefSeq ID: NC_008525.1] For correct ordering and orientation using MeDuSa WebServer: http://combo.dbe.unifi.it/medusa, Accessed February 2, 2023 (Bosi et al., 2015) To generate a final draft genome. Gene prediction and annotation Are performed using The online subsystem-based bacterial genome annotation server RAST: https://rast.nmpdr.org/, Accessed February 7, 2023 (Aziz et al., 2008). Ribosomal RNAs (rRNAs) and transfer RNAs (tRNAs) were detected using barrnap (Seemann, 2023) and ARAGORN (Laslett and Canback, 2004) tools, respectively.

### 2.3 Phylogenetic analysis and genome visualization

Phylogenetic analysis was performed using the 16 s rRNA gene to study the evolutionary relationship of the DSPZPP1 strain with other *Pediococcus* strains, obtaining dendrograms by the unweighted pair group method (UPGMA) clustering algorithm. 16 s rRNA gene sequence of DSPZPP1 was screened against the ‘16 s rRNA sequence database’ in EZBioCloud: https://www.ezbiocloud.net/, Accessed February 9, 2023 (Yoon et al., 2017a). Multiple sequence alignment (MSA) was performed using the MUSCLE program (Edgar, 2004) in MEGA11 standalone software (Tamura et al., 2021). Output MEGA alignment file was used to construct the phylogeny tree using the neighbor-joining (Mailund et al., 2006) method with a bootstrap value of 500. Graphical visualization of different sequence features for the circular genome of *P. pentosaceus* DSPZPP1 was performed using CGView’s PROKSEE server: https://proksee.ca/, Accessed February 9, 2023 (Stothard et al., 2019).

### 2.4 Comparative genome analysis

To evaluate the similarity between the DSPZPP1 draft genome and other *Pediococcus* strains, a pairwise genome sequence comparison was performed. This was measured in terms of average nucleotide identity (ANI) (Yoon et al., 2017b), and correlation indexes of tetra-nucleotide signatures (Tetra) (Noble et al., 1998) were studied by comparing the draft genome sequence of DSPZPP1 strain against complete genome sequences of other *Pediococcus* strains published on NCBI. Average nucleotide identity was calculated using BLAST (ANIb) (McGinnis and Madden, 2004) and MUMMER (ANIm) (Marçais et al., 2018), and analysis was performed using JSpeciesWS: https://jspecies.ribohost.com/jspeciesws/, Accessed February 9, 2023 (Richter et al., 2016).

### 2.5 Identification of plasmids and bacteriophages

DSPZPP1 Draft genome was screened for presence of plasmids and bacteriophages using PlasmidFinder v2.1: https://cge.food.dtu.dk/services/PlasmidFinder/, Accessed February 7, 2023 (Carattoli et al., 2014) and PHAge Search Tool Enhanced Release PHASTER webserver: https://phaster.ca/, Accessed February 9, 2023 (Arndt et al., 2016), respectively.

### 2.6 Identification of antimicrobial resistance genes, virulence genes, and prediction of pathogenicity

Screening of antimicrobial resistance genes was performed against different databases including Comprehensive Antibiotic Resistance Database (CARD) (Alcock et al., 2023), ResFinder (Florensa et al., 2022), ARG_ANNOT (Gupta et al., 2014), and NCBI AMRFinder Plus (Feldgarden et al., 2021). In addition, virulence factor genes in the draft genome were identified using the Virulence Factor Database (VFDB; Chen et al., 2005). Screening of both antimicrobial resistance and virulence genes against different mentioned databases is performed using the standalone tool ABRicate.^2^ In addition, the pathogenicity of sequenced strain toward the human host is assessed using PathogenFinder v1.1: https://cge.food.dtu.dk/services/PathogenFinder/, Accessed February 9, 2023 (Cosentino et al., 2013).

### 2.7 Analysis of carbohydrate-active enzymes (CAZymes)

Identification of different classes of carbohydrate active enzymes (CAZymes) was performed using an HMMER-based search (Wheeler and Eddy, 2013) against the carbohydrate-active enzyme database (CAZyDB) (Drula et al., 2022). Draft genome assembly of DSPZPP1 was scanned in dbCAN3 webserver: https://bcb.unl.edu/dbCAN2/index.php, Accessed February 13, 2023 (Yin et al., 2012) for automated annotation of CAZymes with E-value and coverage cutoff of 1e-15 and 0.35, respectively.

### 2.8 Identification of bacteriocins

Bacteriocins in the DSPZPP1 draft genome were analyzed using the web tool BAGEL4: http://bagel4.molgenrug.nl/, Accessed February 14, 2023 (van Heel et al., 2018). Subsequently, the bacteriocin domains were manually confirmed using BLASTp: https://blast.ncbi.nlm.nih.gov/Blast.cgi, Accessed February 14, 2023 (McGinnis and Madden, 2004) against databases of non-redundant protein sequences (nr) with 30% identity and 80% coverage thresholds.

## 3 Results

### 3.1 Draft genome assembly characteristics of *Pediococcus pentosaceus* DSPZPP1

Whole-genome sequencing using Illumina NextSeq 500 generated approximately 6.14 million paired-end reads. After quality control analysis, approximately 5.15 million HQ reads were obtained which were assembled into 15 scaffolds to generate the final draft genome of size 1,891,324 bp, with a G + C content of 37.3%. A total of 1988 protein-coding RNA genes were identified. Detailed genome assembly statistics and other characteristics are described in Table 1.

**Table 1:** Assembly statistics and genome characteristics of *P. pentosaceus* DSPZPP1.

Attribute	Value
Total no. of sequences	15
Size (bp)	1,891,398
Minimum sequence length (bp)	211
Maximum sequence length (bp)	1,848,864
Median sequence length (bp)	876
Mean sequence length (bp)	126093.2
N50 length (bp)	1,848,864
L50	3
GC content (%)	37.30
Count of protein-coding genes	1930
Count of rRNAs	4 (One 16 s rRNA, one 23 s rRNA and two 5 s rRNAs)
Count of tRNAs	53
Count of tmRNAs	1
Plasmids	0
Bacteriophages	4 (2 intact, 1 incomplete, and 1 questionable)
Probability of being a human pathogen	0.173

### 3.2 Functional annotation

**Figure 1:**
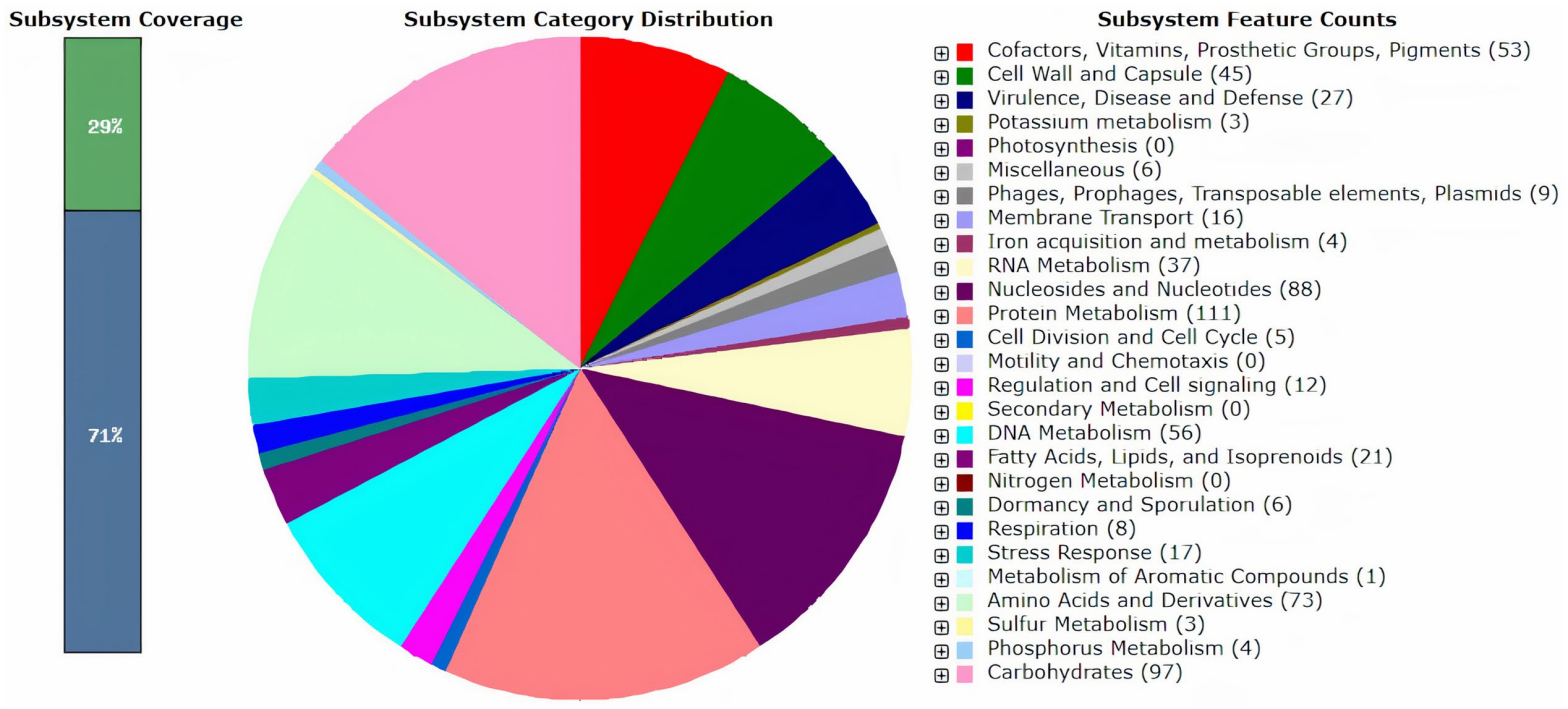
Overview of RAST functional annotation results for the draft genome of *P. pentosaceus* DSPZPP1. The number in parentheses for each subsystem category indicates the count of protein-encoding genes (PEGs) involved in the respective subsystem processes.

Gene prediction and functional annotation of the DSPZPP1 draft genome are performed using RAST^3^ (Aziz et al., 2008), a subsystem-based classification system. A total of 1930 protein-encoding genes (PEGs) and 58 RNAs were predicted [see Supplementary Table S1 (ProteinEncodingGene_Annotation)]. Of 1930 coding sequences, 1,531 were functional (79.32%) and 399 were hypothetical/unknown proteins (20.6%). A total of four ribosomal RNAs, including one 16 s, one 23 s, and two 5 s (one partial sequence), 53 TRNAs, and one tmRNA, were identified. Predicted 1930 protein-encoding genes were assigned to 23 different SEED subsystem categories (Figure 1). It is observed that the maximum pool of protein-encoding genes contributed to “Protein Metabolism” (111) followed by “Carbohydrates” (97) and “Biosynthesis of Nucleosides & Nucleotides” (88). Different LAB strains are known to synthesize water-soluble B-group vitamins, which emphasizes its use in the production of effective vitamin-enriched products (LeBlanc et al., 2011). Our analysis reveals that the DSPZPP1 strain can synthesize B-group vitamins, including biotin, riboflavin, folate, and coenzyme A [see Supplementary Table S1 (RASTDetailedSubsystemAnnotation)], making DSPZPP1, a stronger probiotic candidate.

### 3.3 Taxonomy analysis and genome visualization

**Figure 2:**
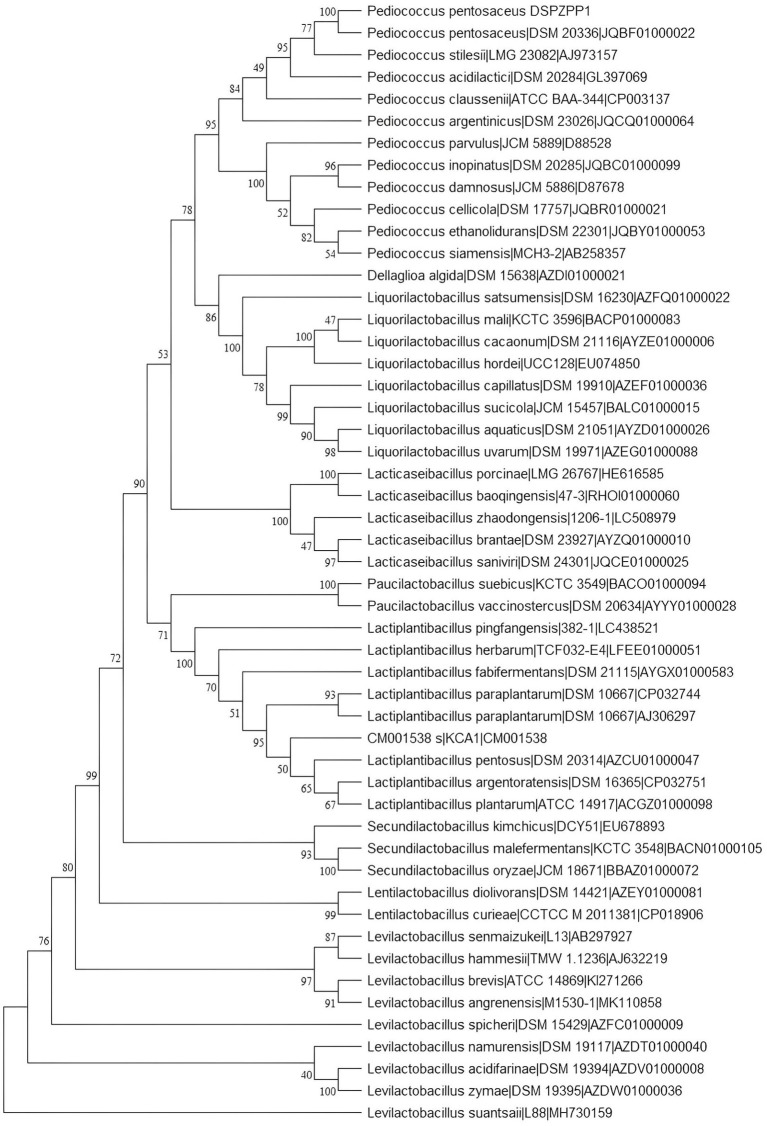
16s rRNA gene-based phylogeny tree constructed using MEGA11. All the *Pediococcus* strains form a separate branch confirming the closest evolutionary relationship. The DSPZPP1 strain is identified as the closest evolutionary neighbor of *P. pentosaceus* DSM 20336.

**Figure 3:**
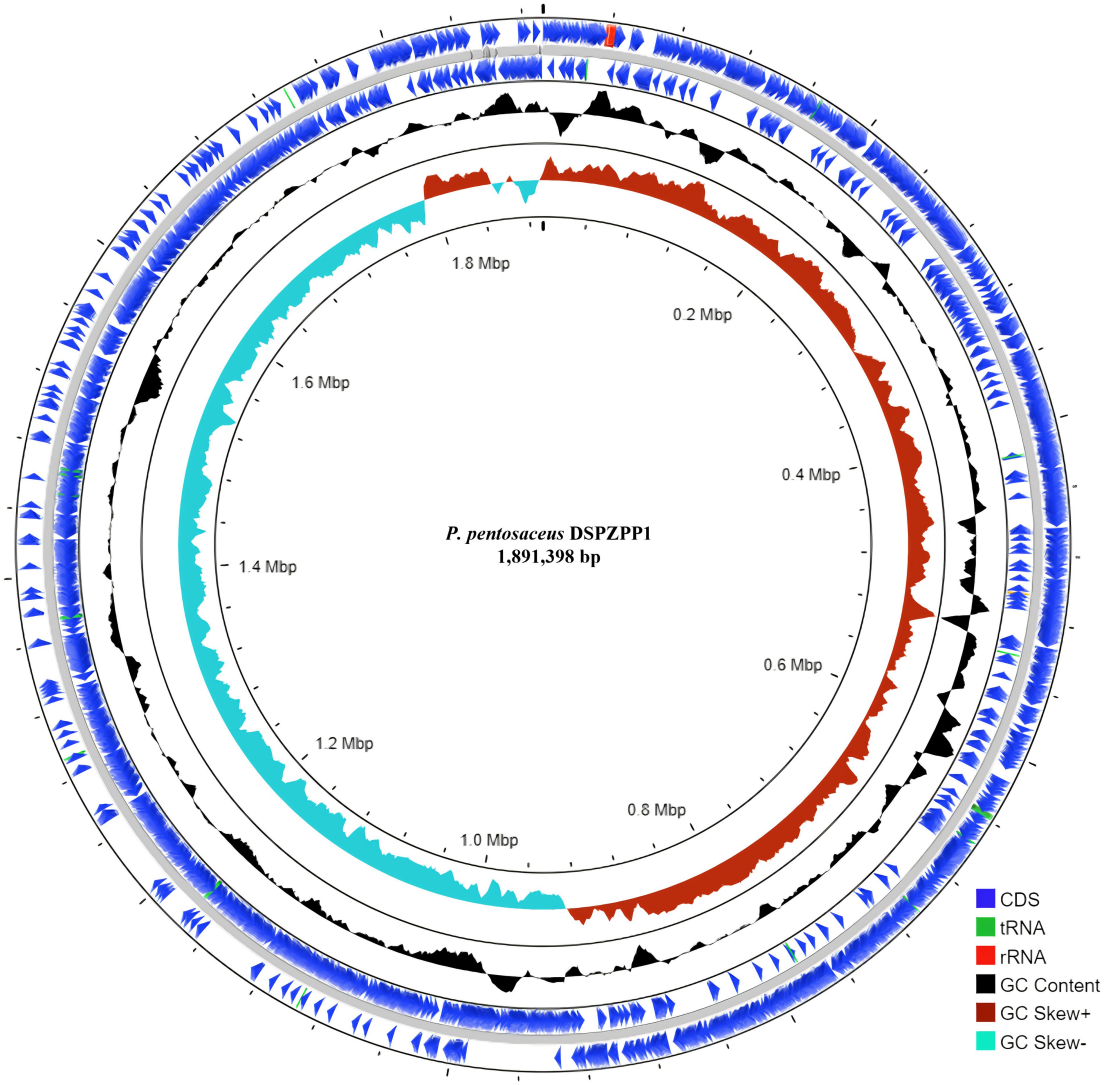
Circular genome map of *P. pentosaceus* DSPZPP1 of size 1.89 Mbp. Genome visualization consists of five rings. The outer concentric circle followed by the second circle represents the position of protein-coding genes and RNAs on forward and reverse strands, respectively. The middle circle represents draft genome scaffolds colored in gray followed by a ring showing GC content (black). Next, the inner ring shows GC Skew [(G–C)/(G+C)] distribution. Positive GC Skew is indicated in red and negative GC skew in sky blue.

The UPGMA tree derived using 16S rRNA gene sequence shows that the DSPZPP1 strain has a closer phylogenetic relationship with other strains from the *Pediococcus* genus. *P. pentosaceus* strains are clustered together forming different branches compared to another genus. The phylogenetic tree derived using MEGA11 (Tamura et al., 2021) is depicted in Figure 2. Phylogeny analysis identified *P. pentosaceus* DSM 20336 strain [NCBI RefSeq accession: NZ_JQBF00000000.1] as the closest homolog of DSPZPP1. The phylogeny tree is depicted in Figure 2. Genomic features of the annotated draft genome of *P. pentosaceus* DSPZPP1 can be visualized using the circular genome map depicted in Figure 3.

### 3.4 Comparative genome analysis

Average nucleotide identity (ANI) analysis using BLAST (McGinnis and Madden, 2004) and MUMMER (Marçais et al., 2018), along with tetra correlation analysis study, confirms *P. pentosaceus* DSM 20336 strain is the closest phylogenetic neighbor of DSPZPP1. This conclusion is supported by the highest tetra correlation coefficient value of 0.9985. The ANI values obtained are 98.54 and 99.04% using BLAST and MUMMER, respectively. Detailed percent identity statistics of this analysis are described in Table 2.

**Table 2:** Average nucleotide identity (ANI) and tetra frequency analysis *P. pentosaceus* DSPZPP1 genome in comparison with other *P. pentosaceus* strains.

Genome	ANIb [%]^a^	%Aligned	ANIm [%]^b^	%Aligned	Tetra correlation coefficient
*P. pentosaceus* DSM 20336	98.54	83.8	99.04	84.41	0.9985
*P. pentosaceus* wikim20	98.44	83.99	98.96	84.55	0.99789
*P. pentosaceus* ATCC 33316	98.54	84.09	99.03	84.52	0.99847
*P. pentosaceus* FDAARGOS_1009	98.55	83.67	99.05	84.18	0.9982
*P. pentosaceus* ATCC 25745	98.3	85.24	98.91	85.56	0.99789
*P. pentosaceus* SL4	98.26	85.77	98.74	86.19	0.99845

^a^Average nucleotide identity calculated using BLAST (ANIb) (McGinnis and Madden, 2004). ^b^Average nucleotide identity calculated using MUMMER (ANIm) (Marçais et al., 2018).

### 3.5 Analysis of antimicrobial resistance (AMR), virulence genes, and pathogenicity

Different LAB strains including *P. pentosaceus* are used in various food industry applications (Lv et al., 2014; Torres-Miranda et al., 2022; Oliveira et al., 2023). Therefore, it is important to conduct a safety assessment prior to its use as a probiotic or within the food industry. Therefore, for the safety evaluation of the DSPZPP1 strain, the draft genome sequence was analyzed for the presence of antimicrobial resistance (AMR) genes, virulence genes, and pathogenicity toward the human host. Analysis shows the absence of antimicrobial resistance (Supplementary File S1) and virulence gene/s. Furthermore, pathogenicity analysis gives a probability value of 0.173, indicating a very low chance of this strain being a human pathogen (Supplementary File S2). Based on these findings, we can safely postulate that the *P. pentosaceus* DSPZPP1 strain is suitable for use as a probiotic and in different food industry applications.

### 3.6 Identification of plasmids and bacteriophages

**Figure 4:**
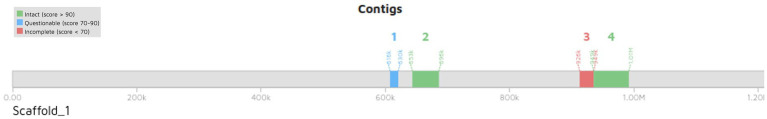
Positions of four identified bacteriophages in draft genome sequence of *P. pentosaceus* DSPZPP1. The four identified bacteriophages include two intact, one questionable, and one incomplete which are located on scaffold 1 having genome coordinates as Scaffold_1:616309 bp - 629709 bp, Scaffold_1:652710 bp −696,134 bp, Scaffold_1:925982–948,780, and Scaffold_1:948804–1,006,219, for bacteriophage one, two, three and four, respectively.

**Figure 5:**
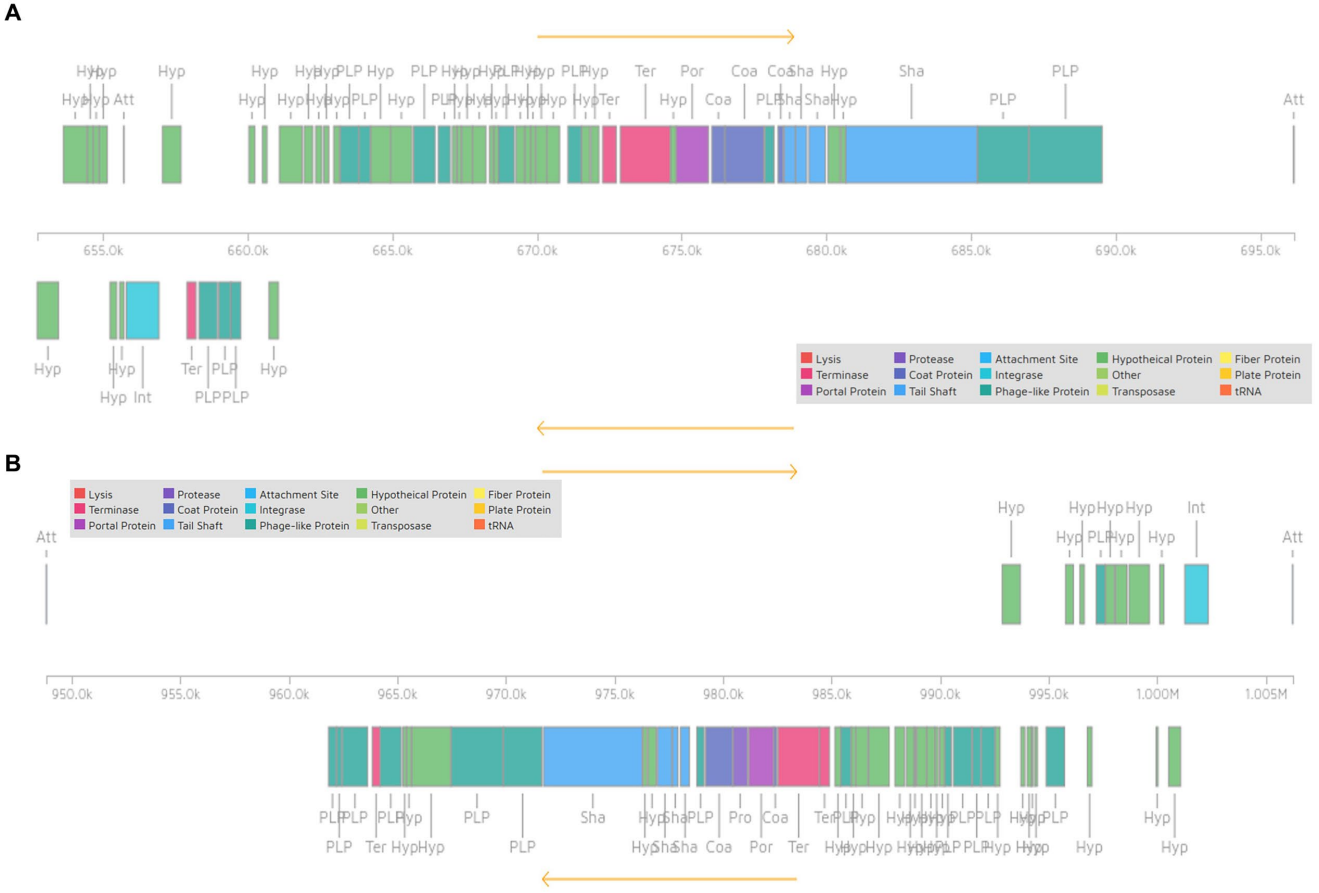
Representation of intact bacteriophage region 1 [Scaffold_1:652710–696,134] **(A)** and region 2 [Scaffold_1:948804–1,006,219] **(B)** showing functional genes.

*Pediococcus pentosaceus* DSPZPP1 does not show the presence of any plasmid sequences. Bacteriophage analysis identified the presence of two intact, one incomplete, and one questionable bacteriophage in the genome. All the identified bacteriophages are located on different regions of scaffold 1 (Figure 4) in the genome. Identified two intact plasmids—region 1 of length 43.4 Kb (Figure 5A) and region 2 of length 57.4 Kb (Figure 5B)—contain significant genes to be functional are described in Table 3. Detailed results of identified bacteriophages are described in Table 3.

**Table 3:** Bacteriophages predicted in draft genome of *P. pentosaceus* DSPZPP1 using PHASTER.

Region	Region length	Completeness	Score	Position in draft genome	GC%	No. of proteins	Most common phage
1	13.4 Kb	Questionable	80	Scaffold_1:616309–629709	39.17%	21	PHAGE_Lactob_phiAT3_NC_005893
2	43.4 Kb	Intact	150	Scaffold_1:652710–696134	36.59%	58	PHAGE_Lactob_Sha1_NC_019489
3	22.7 Kb	Incomplete	40	Scaffold_1:925982–948780	38.07%	12	PHAGE_Bacill_AR9_NC_031039
4	57.4 Kb	Intact	150	Scaffold_1:948804–1006219	37.30%	57	PHAGE_Lactob_Sha1_NC_019489

### 3.7 Analysis of carbohydrate-active enzymes (CAZymes)

**Figure 6:**
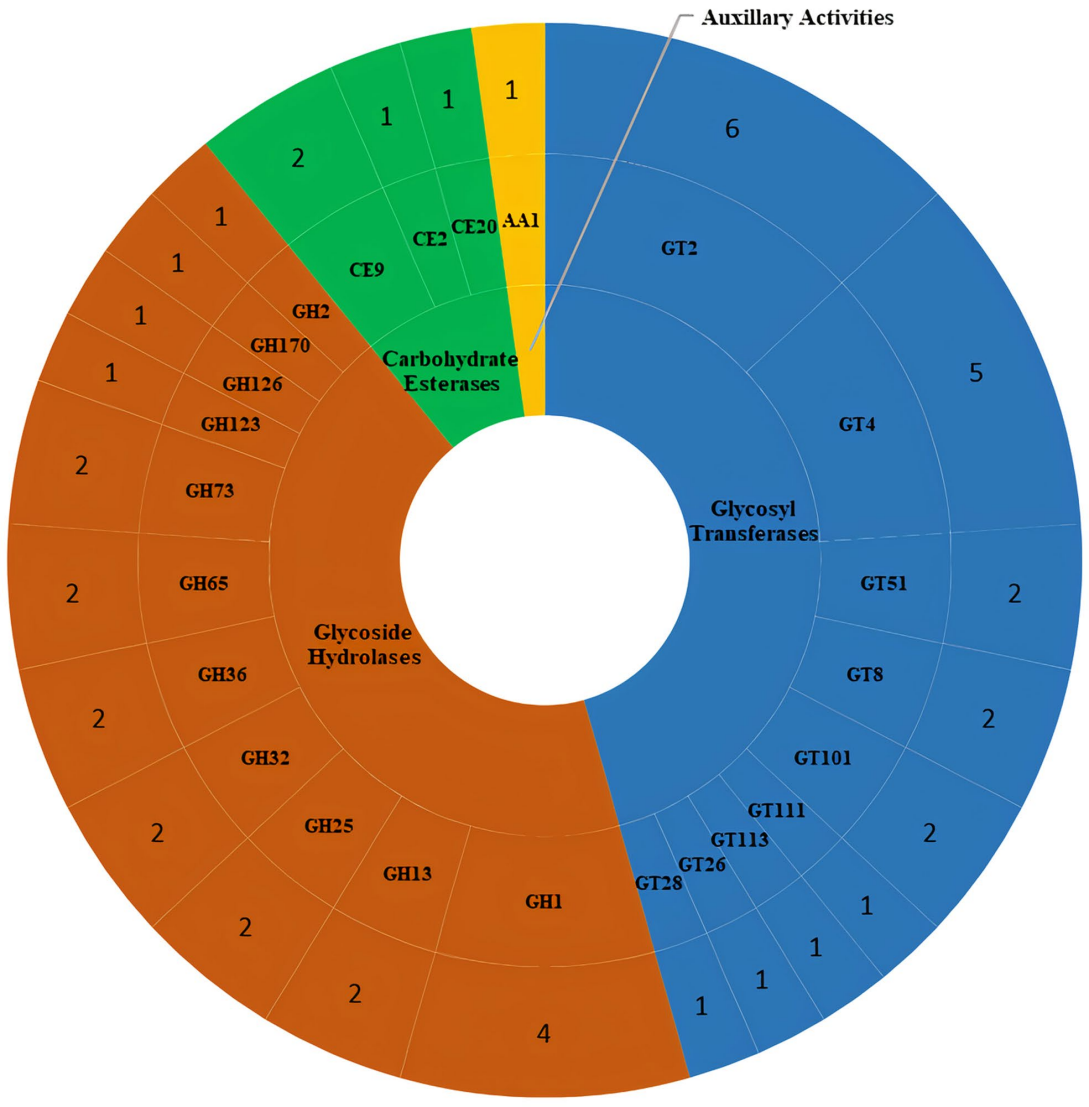
Distribution of different classes of carbohydrate-active enzymes (CAZymes) identified in the *P. pentosaceus* DSPZPP1 draft genome. Different CAZymes classes are represented in different colors. From the inner to the outer ring, the representation includes the Class of CAZyme, CAZyme families, and count of genes identified in each CAZyme family, respectively.

HMMER-based screening in dbCAN3 identified 46 carbohydrate activity-related enzymes in the DSPZPP1 draft genome from four different CAZyme classes, namely glycosyl transferases (GTs), glycoside hydrolases (GHs), carbohydrate esterases (CEs), and auxillary activities (AAs) (Figure 6). Highest number (21) of CAZymes were identified from glycosyl transferases class, which are known to be involved in the formation of glycosidic bonds, followed by glycoside hydrolases (20), modulating the hydrolysis and rearrangement of glycosidic bonds along with few other classes depicted in Figure 6. A list of all identified CAZymes is available in Supplementary Table S2.

### 3.8 Bacteriocin identification

**Figure 7:**
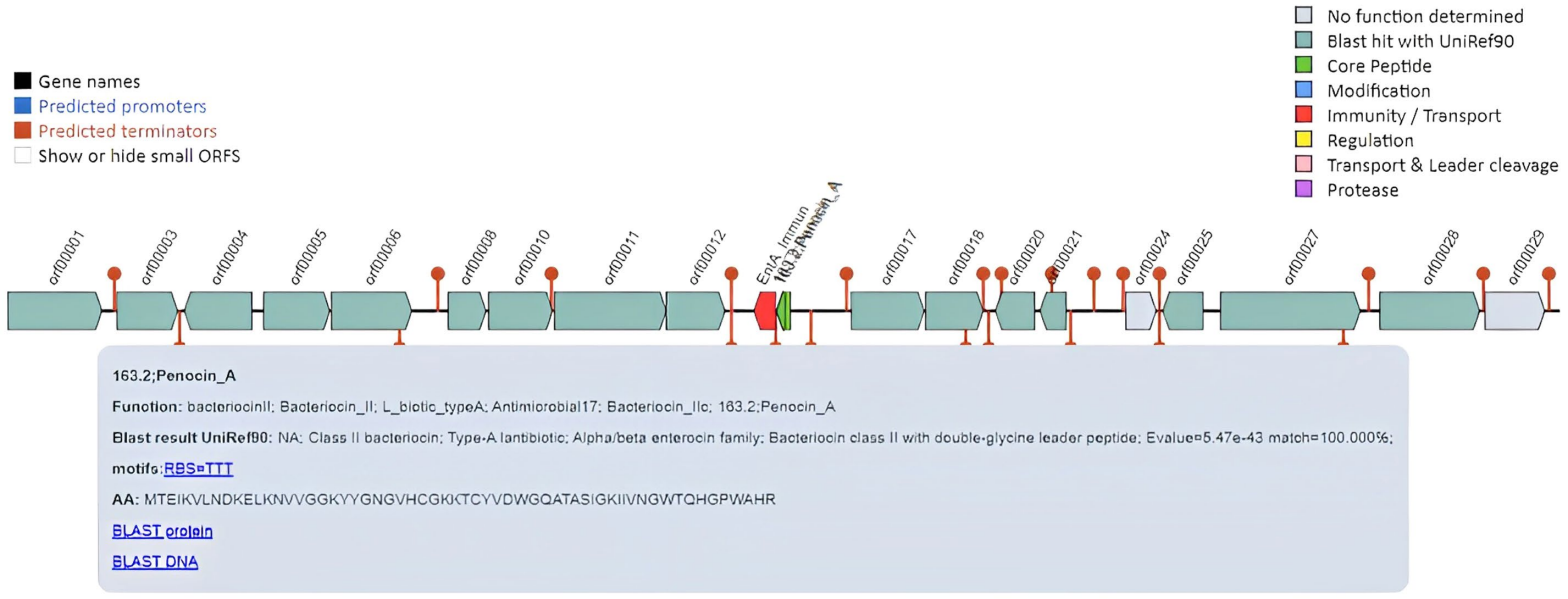
Overview of pediocin-like bacteriocin gene Penocin A in the draft genome of *P. pentosaceus* DSPZPP1.

*Pediococcus pentosaceus* is a lactic acid bacterium, which produces small bactericidal peptides called bacteriocins. *Pediococcus* strains are used as natural preservatives to extend the shelf life and hygienic quality of food because of their characteristic property of producing bacteriocins. It acts against different food contamination-causing bacteria such as *Staphylococcus aureus*, *Listeria monocytogenes*, and *Clostridium botulinum*. DSPZPP1 draft genome was assessed for the presence of bacteriocin compounds. The analysis identified the presence of pediocin-like bacteriocin gene, penocin A, class II bacteriocin (Diep et al., 2006) on scaffold 1 from position 1916 bp to 22,093 bp (Figure 7). Identified bacteriocin peptide sequence is used for BLASTp analysis. Penocin A of the DSPZPP1 genome shows an exact match (100% identity) with the “class II bacteriocin” protein of *Pediococcus* (NCBI Accession ID: WP_011672822.1) having 8e-36 E-value and 100% query coverage. This confirms the presence of bacteriocin penocin A in the draft genome sequence of *P. pentosaceus* DSPZPP1. The analysis concludes the ability of the DSPZPP1 strain to produce the class II bacteriocin, confirming its significance in selection as a probiotic strain.

## 4 Discussion

Currently, numerous *P. pentosaceus* strains have been isolated and identified from different fermented food sources such as milk and sausages. Most of the food-derived strains were shown to be safe and very stable, confirming to have probiotic effects (Min et al., 2017). In the recent years, different *P. pentosaceus* strains were studied (Martino et al., 2013; Vidhyasagar and Jeevaratnam, 2013; Savedboworn et al., 2014; Dubey et al., 2015; Chen et al., 2017) and tested having promising potential as a biological preservative for foods and flavor additive appearing as a potentially predominant probiotic strain (Jiang et al., 2021). It has also been observed that it possesses interesting properties such as antimicrobial ability, resistance to stresses, oxidative level reduction, bile tolerance, β-galactosidase activity, impact on gut microbiome composition, and resistance to gastrointestinal tract conditions (Osmanagaoglu et al., 2010; Dubey et al., 2016; Li et al., 2021). Such investigations support the use of *Pediococcus* strains in the field of probiotics, suggesting role of newly isolated strains from varied food products belonging to the same genus may play a key role in being used in a new generation of functional foods. Looking simultaneously at the continuous demand for safe food by consumers and the unique properties of different LAB strains leading them to potential probiotic candidates, it is necessary to expand the list of potential probiotic bacteria. Therefore, before practical use of bacteria as a probiotic, it is important to perform specific study, which focuses on different aspects involving microorganisms’ safety evaluation, acquired antibiotic resistance, virulence factors, and probiotic viability at the end of shelf life to claim suggested single strain as GRAS (generally regarded as safe) or QPS (qualified presumption of safety) (Dobson et al., 2002; Todorov and Dicks, 2009; Zommiti et al., 2018; Li et al., 2021).

This study conducted on the bacterial strain *P. pentosaceus* DSPZPP1 is the continuation and deepening of previous *in vitro* studies carried out to isolate and investigate the microflora from traditional fermented sausages of Basilicata region, in order to formulate autochthonous starter cultures and to standardize the production process of sausages, to preserve their typical organoleptic and sensory characteristics, and to improve the quality of final product (Parente et al., 2001; Bonomo et al., 2008). In these products, LAB play an important role in meat preservation and fermentation processes and are considered technologically fundamental, providing diversity by the modification of raw material to obtain new sensory properties and improve the safety, stability, and shelf life of meat products (Fontana et al., 2005) and contribute to the flavor, color, and texture development (Aymerich et al., 2006). The identification and technological characterization of LAB involved in meat fermentation are crucial to selecting the best strains to use as starters (Ammor et al., 2005; Rantsiou et al., 2006).

The results of Bonomo et al. (2008) highlighted that the LAB population plays a significant role in influencing the organoleptic and sensory characteristics of the final product. This influence is exerted through specific and important activities that enhance the quality and safety of fermented sausages. Moreover, the LAB ecosystem is characterized by a species-site-dominance, and this dominance is closely related to the environmental parameters. Traditional fermented sausages show a distinctive organoleptic profile, which can be attributed to the isolated strains. Although these strains belong to the species commonly associated with sausage fermentation, they possess specific physiological and technological characteristics that contribute to the uniqueness of these traditional products (Bonomo et al., 2008). Therefore, this study evaluated the complete genome sequence of *P. pentosaceus* DSPZPP1 and performed a complete characterization of the assembled draft genome. Functional annotation of protein-encoding genes predicted from assembled draft genome shows the presence of gene sequences responsible for the metabolism of carbohydrates and proteins, which is an important probiotic and technological feature in LAB (Franz et al., 2006; LeBlanc et al., 2011). Functional annotation analysis highlights the presence of gene sequences responsible for central carbohydrates and metabolism of proteins/amino acids, biosynthesis, and degradation of different compounds, such as cell envelope components, and for transport mechanisms, marking important features in probiotic strains (Oliveira et al., 2023). Moreover, the tolerance to acids and bile was another significant characteristic in LAB because the strains must resist transit and the complexity of the gastrointestinal tract to perform its probiotic functional role. In the *P. pentosaceus* DSPZPP1 genome, the F0F1-ATPase synthase complex proteins and general stress response genes are bacterial defense mechanisms that allow strains to respond and thrive in rapidly changing environmental conditions ensuring the proper functioning of DNA repair and structure pathways (Cotter and Hill, 2003; Sanchez et al., 2008; Oliveira et al., 2023). Functional annotation analysis also revealed the potential of *P. pentosaceus* DSPZPP1 strain to biosynthesize different naturally occurring water-soluble vitamin B molecules including riboflavin, biotin, and folate [see Supplementary Table S1 (RASTDetailedSubsystemAnnotation)].

Many lactic acid bacteria (LAB) strains such as *L. fermentum*, *L. gasseri*, and *L. lactis* are known to produce B-group vitamins (Sybesma et al., 2004; Jayashree et al., 2010; LeBlanc et al., 2011). This property makes LAB strains promising supplements to enhance the biological activity of compounds (Nguyen et al., 2015) and are currently being used in different applications in the food industry (LeBlanc et al., 2011; Nguyen et al., 2015; Levit et al., 2021; Wang et al., 2021). The property of vitamin B biosynthesis by isolated DSPZPP1 strain implies its significant potential use as a vitamin supplement along with its other applications in the food industry. 16s rRNA-based phylogeny analysis integrated with ANI and TETRA analysis indicates the closest evolutionary relationship between sequenced *P. pentosaceus* DSPZPP1 strain and other *P. pentosaceus* strains available at NCBI.

In total, 46 CAZymes from four different classes have been identified that play a key role in sugar metabolism. Different types of GTs are involved in the biosynthesis of disaccharides, oligosaccharides, and polysaccharides, which contribute to the formation of glycosidic bonds (Drula et al., 2022). CAZyme analysis identified 21 enzymes belonging to 9 GT families, belonging to GT2 and GT4 families representing 52.4% of the total GTs, which are responsible for the synthesis of sucrose, cellulose and chitin synthases, glucosyltransferase, and galactosyltransferase. Another majority class of enzymes involved in carbohydrate metabolism was that of GHs with an important role in the hydrolysis of the carbohydrate glycosidic bonds; among the 20 GHs belonging to 11 families, the genes responsible for the synthesis of β-glucosidase (GH1), β-galactosidase (GH2), α-glucosidase (GH13) are present in the genome of DSPZPP1 strain. These enzymes are fundamental in the development of the strain in different environments as they act in the metabolism of sugars, such as lactose, sucrose, and oligosaccharides (Vera et al., 2020; Drula et al., 2022; Oliveira et al., 2023). Moreover, enzymes of the family GH73 were also detected, which are responsible for the cleavage of the β-1,4-glycosidic linkage between N-acetylglucosamine (NAG) and N-acetylmuramic acid (NAM) of bacterial peptidoglycan; because of their cleavage specificity, they are commonly described as β-N-acetylglucosaminidases. Enzyme lysozyme (GH25) was also identified as involved in the cleavage of the β-1,4-glycosidic bond of bacterial cell walls. The characterized lysozymes from this family exhibit both β-1,4-N-acetyl- and β-1,4-N,6-O-diacetylmuramidase activities and degrade O-acetylated peptidoglycan present in *Staphylococcus aureus* and other pathogens (Levit et al., 2021). These enzymes work as a primary line of defense against bacterial infections. Additionally, the *P. pentosaceus* DSPZPP1 genome also encodes GH36 enzymes that exhibit α-galactosidase and α-N-acetylgalactosaminidase activity, GH32 family that contains invertases and enzymes hydrolyze fructose-containing different polysaccharides, and enzymes belonging to GH65 act on substrates containing α-glucosidic linkages and contains mainly phosphorylases (Vera et al., 2020). The genome-encoded CAZymes play an important role in carbohydrate synthesis and hydrolysis during the fermentation process; so, the ability of the bacteria to use carbohydrates is a significant indicator of the functionality of the strain and puts the basics for the cultivation and selection of the strain (Jiang et al., 2020). A further class of enzymes identified with the CAZymes analysis was CEs belonging to three families. CE2 enzymes are α/β-hydrolases, containing an N-terminal β-sheet “jelly-roll” domain that acts as a carbohydrate-binding domain (CBM) and is linked to a C-terminal domain that contains the α/β-hydrolase fold (SGNH-hydrolase motif) (Vollmer, 2008). CE family 9 esterases catalyze the deacetylation of N-acetylglucosamine-6-phosphate to glucosamine-6-phosphate and this reaction has been demonstrated to be important for both amino sugar metabolism and peptidoglycan cell wall recycling in bacteria (Egloff et al., 2001). Carbohydrate esterase family 20 (CE20) comprises xyloglucan acetyl esterases (XacXaeA) putatively related to arabinoxylan deacetylation. XacXaeA is the founding member of the family, specific to O-acetylation, as it is not capable of cleaving N-acetylated carbohydrates. It shows activity on a broad range of O-acetylated mono- and disaccharides and did not show a positional preference for acetylated oxygens. XacXaeA was active toward cell wall extracted xyloglucan oligosaccharides, deacetylating distinct types of structures (Montanier et al., 2009).

The effective probiotic LAB strains mediate antagonistic effects against pathogens by producing internal antimicrobial substances, influencing the metabolism, or stimulating the host immunity. These strains can tolerate acid and bile, adhere to epithelial surfaces, and exhibit antimicrobial properties and antagonistic activity against intestinal pathogens (Park, 2001; Liu et al., 2022). Among all effective probiotic LABs, *P. pentosaceus* is a known potential probiotic strain and has shown broad-spectrum antibacterial activity against external foodborne pathogens by preventing the adhesion of pathogenic bacteria in the gut (Bambirra et al., 2007; Savard et al., 2011). *P. pentosaceus* strains have been studied to produce bacteriocins or other antimicrobial compounds, such as extracellular polysaccharides, when they form biofilms or are attached to the surface, and have been found to show antagonistic effects against various foodborne pathogens (Papagianni and Anastasiadou, 2009; Ladha and Jeevaratnam, 2018; Todhanakasem et al., 2020). In our analysis, the *P. pentosaceus* DSPZPP1 genome shows the presence of the pediocin-like bacteriocin gene penocin A, a class II bacteriocin. Class II pediocin-like bacteriocins are an important and dominant group of antimicrobial peptides that have common features in gene clusters, the structural genes coding for the central peptide, the gene coding for the immune protein and the transporter genes (Papagianni and Anastasiadou, 2009; Cui et al., 2012; Collins et al., 2017; Chen et al., 2020; Tatsaporn and Kornkanok, 2020; Oliveira et al., 2023). In this study, the identified operon of penocin A presented the necessary genes and it is a cluster of biosynthetic genes encoding bacteriocin (Miller et al., 2005; Drider et al., 2006).

In addition to this probiotic characteristic, the draft genome does not show the presence of virulence genes and antimicrobial resistance (AMR) genes. Low pathogenicity value characterizes sequenced strains as non-human pathogens and can be safely used in biological applications. Therefore, these properties make the *P. pentosaceus* DSPZPP1 strain an excellent candidate to be used safely as a probiotic as well as a natural preservative to improve the quality of food. The presence of mobile genetic elements is an important factor to be investigated in microorganisms destined for food application as the presence of plasmids and prophages can be vehicles of horizontal gene transfer of pathogenic genes or antimicrobial resistance genes to other microorganisms (Canchaya et al., 2003; Ferri et al., 2017; Oliveira et al., 2023). The absence of plasmid sequences indicates the stability of the DSPZPP1 genome. Four bacteriophages were identified located at different positions in the genome described in Table 3.

In conclusion, our analysis revealed that the *P. pentosaceus* DSPZPP1 strain, isolated from Italian fermented sausages, can serve as a promising probiotic bacterium. Additionally, it may be used as a future additive in biopreservation to enhance food quality, extend shelf life, and contribute to the final technological properties of the biological product. Furthermore, detailed elucidation of the DSPZPP1 draft genome sequence provides deeper insights into the characteristics of isolated strains, which may contribute to the development of probiotics and future additives in future.

## Data availability statement

The datasets presented in this study can be found in online repositories. The names of the repository/repositories and accession number(s) can be found in the article/Supplementary material.

## Author contributions

MT: Investigation, Writing – original draft, Data curation, Formal analysis. MGB: Investigation, Writing – original draft, Conceptualization, Methodology. SZ: Conceptualization, Methodology, Writing – original draft. SM: Investigation, Writing – original draft. MB: Formal analysis, Methodology, Writing – original draft. IC: Supervision, Writing – review & editing. MC: Project administration, Supervision, Validation, Writing – review & editing. GS: Project administration, Supervision, Validation, Writing – review & editing.

## Footnotes

^1^ https://www.bioinformatics.babraham.ac.uk/projects/fastqc/

^2^ https://github.com/tseemann/abricate

^3^ https://rast.nmpdr.org/rast.cgi
