# Supplementary material for: Whole-genome analysis suggesting probiotic potential and safety properties of Pediococcus pentosaceus DSPZPP1, a promising LAB strain isolated from traditional fermented sausages of the Basilicata region (Southern Italy)
Source: Front Microbiol. 2024 Apr 4;15:1268216. doi: 10.3389/fmicb.2024.1268216 (PMC11024341; doi:10.3389/fmicb.2024.1268216)
Supplement: Supplementary file 4 [file Table_4.DOCX]

INPUT SEQUENCE INFO:

Input Name:: 4_02_02_2023_215019_504536_input.fsa

Sequences:: 1930

Total bpp:: 559830

Longest seq:: 1666

Shortest seq:: 32

Avg seq lenght :: 290.0

RESULTS:

Min Identity Threshold ::100.0

Z-THRESHOLD:: 3.0

Prediction Score:: -228.652

Probability of being human pathogen: 0.173

Matches:: 105

Genome Coverage (%):: 5.44

Pathogenic Families Matched:: 0

Non-Pathogenic Families Matched :: 105

The organisms is predicted as human pathogenic :: No

MATCHED SEQUENCES:

#input_seq: fig|1255.481.peg.346

#matched_seq: 398 CP000422 Pediococcus pentosaceus ATCC 25745, complete genome. Lactobacillales Superfamily I DNA and RNA helicase ABJ67500 No 100.0

#input_seq: fig|1255.481.peg.123

#matched_seq: 398 CP000422 Pediococcus pentosaceus ATCC 25745, complete genome. Lactobacillales Na+/xyloside symporter related transporter ABJ67304 No 100.0

#input_seq: fig|1255.481.peg.589

#matched_seq: 398 CP000422 Pediococcus pentosaceus ATCC 25745, complete genome. Lactobacillales Na+/xyloside symporter related transporter ABJ67604 No 100.0

#input_seq: fig|1255.481.peg.1130

#matched_seq: 398 CP000422 Pediococcus pentosaceus ATCC 25745, complete genome. Lactobacillales Predicted hydrolase of the metallo-beta-lactamase superfamily ABJ68199 No 100.0

#input_seq: fig|1255.481.peg.1576

#matched_seq: 398 CP000422 Pediococcus pentosaceus ATCC 25745, complete genome. Lactobacillales Chloride channel protein EriC ABJ68643 No 100.0

#input_seq: fig|1255.481.peg.289

#matched_seq: 398 CP000422 Pediococcus pentosaceus ATCC 25745, complete genome. Lactobacillales Hemolysins related protein with CBS domains ABJ67442 No 100.0

#input_seq: fig|1255.481.peg.1596

#matched_seq: 398 CP000422 Pediococcus pentosaceus ATCC 25745, complete genome. Lactobacillales Uncharacterized NAD(FAD)-dependent dehydrogenase ABJ68662 No 100.0

#input_seq: fig|1255.481.peg.1534

#matched_seq: 398 CP000422 Pediococcus pentosaceus ATCC 25745, complete genome. Lactobacillales Na+-driven multidrug efflux pump ABJ68601 No 100.0

#input_seq: fig|1255.481.peg.1140

#matched_seq: 398 CP000422 Pediococcus pentosaceus ATCC 25745, complete genome. Lactobacillales cell division membrane protein ABJ68209 No 100.0

#input_seq: fig|1255.481.peg.1818

#matched_seq: 398 CP000422 Pediococcus pentosaceus ATCC 25745, complete genome. Lactobacillales permease of the major facilitator superfamily ABJ67124 No 100.0

#input_seq: fig|1255.481.peg.1589

#matched_seq: 398 CP000422 Pediococcus pentosaceus ATCC 25745, complete genome. Lactobacillales permease of the major facilitator superfamily ABJ68656 No 100.0

#input_seq: fig|1255.481.peg.1675

#matched_seq: 398 CP000422 Pediococcus pentosaceus ATCC 25745, complete genome. Lactobacillales Signal transduction histidine kinase ABJ68737 No 100.0

#input_seq: fig|1255.481.peg.533

#matched_seq: 398 CP000422 Pediococcus pentosaceus ATCC 25745, complete genome. Lactobacillales Predicted permease ABJ67688 No 100.0

#input_seq: fig|1255.481.peg.311

#matched_seq: 398 CP000422 Pediococcus pentosaceus ATCC 25745, complete genome. Lactobacillales Predicted multitransmembrane protein ABJ67465 No 100.0

#input_seq: fig|1255.481.peg.92

#matched_seq: 398 CP000422 Pediococcus pentosaceus ATCC 25745, complete genome. Lactobacillales transcriptional regulator, GntR family ABJ67264 No 100.0

#input_seq: fig|1255.481.peg.1824

#matched_seq: 398 CP000422 Pediococcus pentosaceus ATCC 25745, complete genome. Lactobacillales ABC-type antimicrobial peptide transport system, permease component ABJ67129 No 100.0

#input_seq: fig|1255.481.peg.1153

#matched_seq: 398 CP000422 Pediococcus pentosaceus ATCC 25745, complete genome. Lactobacillales Predicted permease ABJ68221 No 100.0

#input_seq: fig|1255.481.peg.251

#matched_seq: 398 CP000422 Pediococcus pentosaceus ATCC 25745, complete genome. Lactobacillales Auxin efflux carrier (AEC) family permease ABJ67406 No 100.0

#input_seq: fig|1255.481.peg.172

#matched_seq: 398 CP000422 Pediococcus pentosaceus ATCC 25745, complete genome. Lactobacillales Exopolyphosphatase ABJ67354 No 100.0

#input_seq: fig|1255.481.peg.1603

#matched_seq: 398 CP000422 Pediococcus pentosaceus ATCC 25745, complete genome. Lactobacillales permease of the drug/metabolite transporter (DMT) superfamily ABJ68668 No 100.0

#input_seq: fig|1255.481.peg.321

#matched_seq: 398 CP000422 Pediococcus pentosaceus ATCC 25745, complete genome. Lactobacillales prolyl aminopeptidase ABJ67475 No 100.0

#input_seq: fig|1255.481.peg.17

#matched_seq: 398 CP000422 Pediococcus pentosaceus ATCC 25745, complete genome. Lactobacillales ABC-type metal ion transport system, periplasmic component/surface adhesin ABJ67197 No 100.0

#input_seq: fig|1255.481.peg.1574

#matched_seq: 398 CP000422 Pediococcus pentosaceus ATCC 25745, complete genome. Lactobacillales Aryl-alcohol dehydrogenase related enzyme ABJ68641 No 100.0

#input_seq: fig|1255.481.peg.1336

#matched_seq: 398 CP000422 Pediococcus pentosaceus ATCC 25745, complete genome. Lactobacillales Restriction endonuclease ABJ68403 No 100.0

#input_seq: fig|1255.481.peg.1100

#matched_seq: 398 CP000422 Pediococcus pentosaceus ATCC 25745, complete genome. Lactobacillales hypothetical protein ABJ68169 No 100.0

#input_seq: fig|1255.481.peg.1262

#matched_seq: 398 CP000422 Pediococcus pentosaceus ATCC 25745, complete genome. Lactobacillales rod shape-determining protein MreC ABJ68329 No 100.0

#input_seq: fig|1255.481.peg.332

#matched_seq: 398 CP000422 Pediococcus pentosaceus ATCC 25745, complete genome. Lactobacillales ABC-type amino acid transport/signal transduction system, periplasmic component/domain ABJ67486 No 100.0

#input_seq: fig|1255.481.peg.1334

#matched_seq: 398 CP000422 Pediococcus pentosaceus ATCC 25745, complete genome. Lactobacillales Transcriptional regulator ABJ68402 No 100.0

#input_seq: fig|1255.481.peg.118

#matched_seq: 398 CP000422 Pediococcus pentosaceus ATCC 25745, complete genome. Lactobacillales mannose-specific PTS system component IID ABJ67301 No 100.0

#input_seq: fig|1255.481.peg.1540

#matched_seq: 398 CP000422 Pediococcus pentosaceus ATCC 25745, complete genome. Lactobacillales hypothetical protein ABJ68607 No 100.0

#input_seq: fig|1255.481.peg.588

#matched_seq: 398 CP000422 Pediococcus pentosaceus ATCC 25745, complete genome. Lactobacillales transcriptional regulator, AraC family ABJ67603 No 100.0

#input_seq: fig|1255.481.peg.254

#matched_seq: 398 CP000422 Pediococcus pentosaceus ATCC 25745, complete genome. Lactobacillales Predicted hydrolase of the HAD superfamily ABJ67408 No 100.0

#input_seq: fig|1255.481.peg.1561

#matched_seq: 398 CP000422 Pediococcus pentosaceus ATCC 25745, complete genome. Lactobacillales ABC-type polysaccharide/polyol phosphate export system, permease component ABJ68628 No 100.0

#input_seq: fig|1255.481.peg.149

#matched_seq: 398 CP000422 Pediococcus pentosaceus ATCC 25745, complete genome. Lactobacillales Hydroxymethylpyrimidine/phosphomethylpyrimidine kinase ABJ67329 No 100.0

#input_seq: fig|1255.481.peg.874

#matched_seq: 398 CP000422 Pediococcus pentosaceus ATCC 25745, complete genome. Lactobacillales Mn-containing catalase ABJ67964 No 100.0

#input_seq: fig|1255.481.peg.286

#matched_seq: 398 CP000422 Pediococcus pentosaceus ATCC 25745, complete genome. Lactobacillales protein tyrosine phosphatase ABJ67439 No 100.0

#input_seq: fig|1255.481.peg.203

#matched_seq: 398 CP000422 Pediococcus pentosaceus ATCC 25745, complete genome. Lactobacillales ABC-type Mn2+/Zn2+ transport system, permease component ABJ67388 No 100.0

#input_seq: fig|1255.481.peg.317

#matched_seq: 398 CP000422 Pediococcus pentosaceus ATCC 25745, complete genome. Lactobacillales methionine aminopeptidase, type I ABJ67471 No 100.0

#input_seq: fig|1255.481.peg.1550

#matched_seq: 398 CP000422 Pediococcus pentosaceus ATCC 25745, complete genome. Lactobacillales hypothetical protein ABJ68617 No 100.0

#input_seq: fig|1255.481.peg.312

#matched_seq: 398 CP000422 Pediococcus pentosaceus ATCC 25745, complete genome. Lactobacillales Predicted multitransmembrane protein ABJ67466 No 100.0

#input_seq: fig|1255.481.peg.403

#matched_seq: 398 CP000422 Pediococcus pentosaceus ATCC 25745, complete genome. Lactobacillales triosephosphate isomerase ABJ67556 No 100.0

#input_seq: fig|1255.481.peg.295

#matched_seq: 398 CP000422 Pediococcus pentosaceus ATCC 25745, complete genome. Lactobacillales transcriptional regulator, GntR family ABJ67448 No 100.0

#input_seq: fig|1255.481.peg.1860

#matched_seq: 398 CP000422 Pediococcus pentosaceus ATCC 25745, complete genome. Lactobacillales Uncharacterized ABC transporter, ATPase component ABJ67164 No 100.0

#input_seq: fig|1255.481.peg.1520

#matched_seq: 398 CP000422 Pediococcus pentosaceus ATCC 25745, complete genome. Lactobacillales Sortase (surface protein transpeptidase) ABJ68587 No 100.0

#input_seq: fig|1255.481.peg.202

#matched_seq: 398 CP000422 Pediococcus pentosaceus ATCC 25745, complete genome. Lactobacillales ABC-type Mn/Zn transport system, ATPase component ABJ67387 No 100.0

#input_seq: fig|1255.481.peg.1842

#matched_seq: 398 CP000422 Pediococcus pentosaceus ATCC 25745, complete genome. Lactobacillales C-di-GMP-specific phosphodiesterase ABJ67146 No 100.0

#input_seq: fig|1255.481.peg.1598

#matched_seq: 398 CP000422 Pediococcus pentosaceus ATCC 25745, complete genome. Lactobacillales C-di-GMP-specific phosphodiesterase ABJ68663 No 100.0

#input_seq: fig|1255.481.peg.343

#matched_seq: 398 CP000422 Pediococcus pentosaceus ATCC 25745, complete genome. Lactobacillales Fructose-2,6-bisphosphatase ABJ67497 No 100.0

#input_seq: fig|1255.481.peg.1650

#matched_seq: 398 CP000422 Pediococcus pentosaceus ATCC 25745, complete genome. Lactobacillales cAMP-binding protein - catabolite gene activator and regulatory subunit of cAMP-dependent protein kinase ABJ68716 No 100.0

#input_seq: fig|1255.481.peg.1436

#matched_seq: 398 CP000422 Pediococcus pentosaceus ATCC 25745, complete genome. Lactobacillales Acetyltransferase ABJ68504 No 100.0

#input_seq: fig|1255.481.peg.724

#matched_seq: 398 CP000422 Pediococcus pentosaceus ATCC 25745, complete genome. Lactobacillales Dephospho-CoA kinase ABJ67751 No 100.0

#input_seq: fig|1255.481.peg.532

#matched_seq: 398 CP000422 Pediococcus pentosaceus ATCC 25745, complete genome. Lactobacillales Uncharacterized domain/protein associated with RNase G and E ABJ67687 No 100.0

#input_seq: fig|1255.481.peg.920

#matched_seq: 398 CP000422 Pediococcus pentosaceus ATCC 25745, complete genome. Lactobacillales hypothetical protein ABJ67991 No 100.0

#input_seq: fig|1255.481.peg.910

#matched_seq: 398 CP000422 Pediococcus pentosaceus ATCC 25745, complete genome. Lactobacillales Predicted integral membrane protein ABJ67981 No 100.0

#input_seq: fig|1255.481.peg.1294

#matched_seq: 398 CP000422 Pediococcus pentosaceus ATCC 25745, complete genome. Lactobacillales F0F1-type ATP synthase, delta subunit ABJ68361 No 100.0

#input_seq: fig|1255.481.peg.745

#matched_seq: 398 CP000422 Pediococcus pentosaceus ATCC 25745, complete genome. Lactobacillales Predicted metal-binding, possibly nucleic acid-binding protein ABJ67772 No 100.0

#input_seq: fig|1255.481.peg.776

#matched_seq: 398 CP000422 Pediococcus pentosaceus ATCC 25745, complete genome. Lactobacillales dUTPase ABJ67803 No 100.0

#input_seq: fig|1255.481.peg.1845

#matched_seq: 398 CP000422 Pediococcus pentosaceus ATCC 25745, complete genome. Lactobacillales Acetyltransferase, GNAT family ABJ67149 No 100.0

#input_seq: fig|1255.481.peg.1467

#matched_seq: 398 CP000422 Pediococcus pentosaceus ATCC 25745, complete genome. Lactobacillales deoxyuridine 5'-triphosphate nucleotidohydrolase ABJ68535 No 100.0

#input_seq: fig|1255.481.peg.293

#matched_seq: 398 CP000422 Pediococcus pentosaceus ATCC 25745, complete genome. Lactobacillales B ABJ67446 No 100.0

#input_seq: fig|1255.481.peg.247

#matched_seq: 398 CP000422 Pediococcus pentosaceus ATCC 25745, complete genome. Lactobacillales hypothetical protein ABJ67402 No 100.0

#input_seq: fig|1255.481.peg.37

#matched_seq: 398 CP000422 Pediococcus pentosaceus ATCC 25745, complete genome. Lactobacillales hypothetical protein ABJ67214 No 100.0

#input_seq: fig|1255.481.peg.1407

#matched_seq: 398 CP000422 Pediococcus pentosaceus ATCC 25745, complete genome. Lactobacillales hypothetical protein ABJ68474 No 100.0

#input_seq: fig|1255.481.peg.1060

#matched_seq: 398 CP000422 Pediococcus pentosaceus ATCC 25745, complete genome. Lactobacillales purine deoxyribosyltransferase ABJ68130 No 100.0

#input_seq: fig|1255.481.peg.1314

#matched_seq: 398 CP000422 Pediococcus pentosaceus ATCC 25745, complete genome. Lactobacillales transcription elongation factor GreA ABJ68381 No 100.0

#input_seq: fig|1255.481.peg.186

#matched_seq: 398 CP000422 Pediococcus pentosaceus ATCC 25745, complete genome. Lactobacillales Universal stress protein UspA related nucleotide-binding protein ABJ67371 No 100.0

#input_seq: fig|1255.481.peg.328

#matched_seq: 398 CP000422 Pediococcus pentosaceus ATCC 25745, complete genome. Lactobacillales Fe2+/Zn2+ uptake regulation protein ABJ67482 No 100.0

#input_seq: fig|1255.481.peg.1853

#matched_seq: 398 CP000422 Pediococcus pentosaceus ATCC 25745, complete genome. Lactobacillales Universal stress protein UspA related nucleotide-binding protein ABJ67157 No 100.0

#input_seq: fig|1255.481.peg.785

#matched_seq: 398 CP000422 Pediococcus pentosaceus ATCC 25745, complete genome. Lactobacillales hypothetical protein ABJ67875 No 100.0

#input_seq: fig|1255.481.peg.1599

#matched_seq: 398 CP000422 Pediococcus pentosaceus ATCC 25745, complete genome. Lactobacillales heat shock protein Hsp20 ABJ68664 No 100.0

#input_seq: fig|1255.481.peg.325

#matched_seq: 398 CP000422 Pediococcus pentosaceus ATCC 25745, complete genome. Lactobacillales NUDIX family hydrolase ABJ67479 No 100.0

#input_seq: fig|1255.481.peg.1624

#matched_seq: 398 CP000422 Pediococcus pentosaceus ATCC 25745, complete genome. Lactobacillales hypothetical protein ABJ68689 No 100.0

#input_seq: fig|1255.481.peg.831

#matched_seq: 398 CP000422 Pediococcus pentosaceus ATCC 25745, complete genome. Lactobacillales 3-hydroxymyristoyl/3-hydroxydecanoyl-(acyl carrier protein) dehydratase ABJ67920 No 100.0

#input_seq: fig|1255.481.peg.1413

#matched_seq: 398 CP000422 Pediococcus pentosaceus ATCC 25745, complete genome. Lactobacillales hypothetical protein ABJ68480 No 100.0

#input_seq: fig|1255.481.peg.1623

#matched_seq: 398 CP000422 Pediococcus pentosaceus ATCC 25745, complete genome. Lactobacillales hypothetical protein ABJ68688 No 100.0

#input_seq: fig|1255.481.peg.731

#matched_seq: 398 CP000422 Pediococcus pentosaceus ATCC 25745, complete genome. Lactobacillales Putative effector of murein hydrolase LrgA ABJ67758 No 100.0

#input_seq: fig|1255.481.peg.1798

#matched_seq: 398 CP000422 Pediococcus pentosaceus ATCC 25745, complete genome. Lactobacillales Diadenosine tetraphosphate (Ap4A) hydrolase related HIT family hydrolase ABJ68859 No 100.0

#input_seq: fig|1255.481.peg.1177

#matched_seq: 398 CP000422 Pediococcus pentosaceus ATCC 25745, complete genome. Lactobacillales cell division protein FtsL ABJ68244 No 100.0

#input_seq: fig|1255.481.peg.1330

#matched_seq: 398 CP000422 Pediococcus pentosaceus ATCC 25745, complete genome. Lactobacillales hypothetical protein ABJ68398 No 100.0

#input_seq: fig|1255.481.peg.581

#matched_seq: 28537 CP001084 Lactobacillus casei str. Zhang, complete genome. Lactobacillales conserved hypothetical protein ADK19588 No 100.0

#input_seq: fig|1255.481.peg.1159

#matched_seq: 398 CP000422 Pediococcus pentosaceus ATCC 25745, complete genome. Lactobacillales hypothetical protein ABJ68227 No 100.0

#input_seq: fig|1255.481.peg.1672

#matched_seq: 398 CP000422 Pediococcus pentosaceus ATCC 25745, complete genome. Lactobacillales Predicted membrane protein ABJ68734 No 100.0

#input_seq: fig|1255.481.peg.292

#matched_seq: 398 CP000422 Pediococcus pentosaceus ATCC 25745, complete genome. Lactobacillales hypothetical protein ABJ67445 No 100.0

#input_seq: fig|1255.481.peg.315

#matched_seq: 398 CP000422 Pediococcus pentosaceus ATCC 25745, complete genome. Lactobacillales Thiol-disulfide isomerase and thioredoxin ABJ67469 No 100.0

#input_seq: fig|1255.481.peg.782

#matched_seq: 398 CP000422 Pediococcus pentosaceus ATCC 25745, complete genome. Lactobacillales Predicted ribosomal protein ABJ67872 No 100.0

#input_seq: fig|1255.481.peg.33

#matched_seq: 398 CP000422 Pediococcus pentosaceus ATCC 25745, complete genome. Lactobacillales Thiol-disulfide isomerase and thioredoxin ABJ67212 No 100.0

#input_seq: fig|1255.481.peg.1341

#matched_seq: 398 CP000422 Pediococcus pentosaceus ATCC 25745, complete genome. Lactobacillales aspartyl/glutamyl-tRNA(Asn/Gln) amidotransferase subunit C ABJ68408 No 100.0

#input_seq: fig|1255.481.peg.739

#matched_seq: 398 CP000422 Pediococcus pentosaceus ATCC 25745, complete genome. Lactobacillales RNA-binding protein, KH domain ABJ67766 No 100.0

#input_seq: fig|1255.481.peg.421

#matched_seq: 398 CP000422 Pediococcus pentosaceus ATCC 25745, complete genome. Lactobacillales hypothetical protein ABJ67574 No 100.0

#input_seq: fig|1255.481.peg.1210

#matched_seq: 398 CP000422 Pediococcus pentosaceus ATCC 25745, complete genome. Lactobacillales Competence protein ComGC ABJ68277 No 100.0

#input_seq: fig|1255.481.peg.277

#matched_seq: 398 CP000422 Pediococcus pentosaceus ATCC 25745, complete genome. Lactobacillales hypothetical protein ABJ67430 No 100.0

#input_seq: fig|1255.481.peg.1124

#matched_seq: 398 CP000422 Pediococcus pentosaceus ATCC 25745, complete genome. Lactobacillales Predicted pyrophosphatase ABJ68193 No 100.0

#input_seq: fig|1255.481.peg.630

#matched_seq: 398 CP000422 Pediococcus pentosaceus ATCC 25745, complete genome. Lactobacillales hypothetical protein ABJ67717 No 100.0

#input_seq: fig|1255.481.peg.1169

#matched_seq: 398 CP000422 Pediococcus pentosaceus ATCC 25745, complete genome. Lactobacillales Cell division membrane protein ABJ68236 No 100.0

#input_seq: fig|1255.481.peg.1162

#matched_seq: 398 CP000422 Pediococcus pentosaceus ATCC 25745, complete genome. Lactobacillales Small membrane protein ABJ68230 No 100.0

#input_seq: fig|1255.481.peg.1428

#matched_seq: 398 CP000422 Pediococcus pentosaceus ATCC 25745, complete genome. Lactobacillales Phosphoribosylformylglycinamidine (FGAM) synthase, PurS component ABJ68495 No 100.0

#input_seq: fig|1255.481.peg.840

#matched_seq: 398 CP000422 Pediococcus pentosaceus ATCC 25745, complete genome. Lactobacillales hypothetical protein ABJ67930 No 100.0

#input_seq: fig|1255.481.peg.867

#matched_seq: 398 CP000422 Pediococcus pentosaceus ATCC 25745, complete genome. Lactobacillales hypothetical protein ABJ67957 No 100.0

#input_seq: fig|1255.481.peg.1146

#matched_seq: 398 CP000422 Pediococcus pentosaceus ATCC 25745, complete genome. Lactobacillales RNA binding protein, RRM domain ABJ68215 No 100.0

#input_seq: fig|1255.481.peg.1709

#matched_seq: 398 CP000422 Pediococcus pentosaceus ATCC 25745, complete genome. Lactobacillales hypothetical protein ABJ68770 No 100.0

#input_seq: fig|1255.481.peg.1729

#matched_seq: 398 CP000422 Pediococcus pentosaceus ATCC 25745, complete genome. Lactobacillales hypothetical protein ABJ68790 No 100.0

#input_seq: fig|1255.481.peg.540

#matched_seq: 398 CP000422 Pediococcus pentosaceus ATCC 25745, complete genome. Lactobacillales hypothetical protein ABJ67695 No 100.0

#input_seq: fig|1255.481.peg.12

#matched_seq: 398 CP000422 Pediococcus pentosaceus ATCC 25745, complete genome. Lactobacillales Prebacteriocin ABJ67193 No 100.0

#input_seq: fig|1255.481.peg.1416

#matched_seq: 398 CP000422 Pediococcus pentosaceus ATCC 25745, complete genome. Lactobacillales hypothetical protein ABJ68483 No 100.0

#input_seq: fig|1255.481.peg.1180

#matched_seq: 398 CP000422 Pediococcus pentosaceus ATCC 25745, complete genome. Lactobacillales hypothetical protein ABJ68247 No 100.0
